# Identification of potential hub genes associated with recurrent miscarriage through combined transcriptomic and proteomic analysis

**DOI:** 10.17305/bb.2024.11158

**Published:** 2024-10-20

**Authors:** Hao-Ran Xu, Long Yang, Yan Gu, Yan Shi, Shu-Han Yang, Jie Gan, Wen-Wen Gu, Xuan Zhang, Jian Wang

**Affiliations:** 1 Shanghai Key Laboratory of Health and Disease Genomics, NHC Key Lab of Reproduction Regulation, Shanghai Institute for Biomedical and Pharmaceutical Technologies, Shanghai, China; 2 The Second Hospital of Tianjin Medical University, Tianjin, China; 3 National Immunological Laboratory of Traditional Chinese Medicine, Affiliated Hospital of Youjiang Medical University for Nationalities, Baise, China

**Keywords:** Recurrent miscarriage, hub genes, decidua, endometrial stromal cells, ISG15

## Abstract

Recurrent miscarriage (RM) is currently difficult to prevent and treat due to a lack of comprehensive understanding of its molecular mechanisms. The aim of this study was to identify genes potentially involved in the pathogenesis of RM and to observe their expression in the decidual tissues of RM patients. A total of 1823 differentially expressed genes (DEGs) and 148 differentially expressed proteins (DEPs) in decidual tissues between RM and control groups were identified. Subsequently, *DCN*, *DPT*, *LUM*, *MFAP4*, and *ISG15* were identified from the DEGs/DEPs as RM-related hub genes through systematic bioinformatics analysis. Bioinformatics analysis of the single-cell dataset GSE214607 revealed that the expression of these five hub genes in the decidual stromal cells (DSCs) of RM patients appeared to be upregulated, while the RT-qPCR assay showed that their decidual expression levels were significantly increased in RM patients. Uterine *Isg15* expression was significantly increased, whereas the uterine expression of *Dcn*, *Dpt*, *Lum*, and *Mfap4* was decreased in LPS-induced early pregnancy loss (EPL) mice. MiR-16-5p, -21-3p, -27a-3p, and -941 were identified as potentially involved in the regulation of these five hub genes, and their decidual expression levels were significantly decreased in RM patients. The abnormally increased ISG15 expression in the decidual tissues of RM patients and uterine tissues of LPS-induced mice was validated by WB analysis. ISG15 expression was significantly reduced during the *in vitro* decidualization of human endometrial stromal cells (hESCs). Collectively, *DCN*, *DPT*, *LUM*, *MFAP4*, and *ISG15* were identified as RM-related hub genes, and their expression in the decidual tissues of RM patients was significantly increased. The decidualization of hESCs was accompanied by reduced ISG15 expression, suggesting that increased decidual ISG15 expression might lead to EPL by disrupting the decidualization process.

## Introduction

Recurrent miscarriage (RM) is a complex pregnancy disorder that affects pregnant women worldwide and causes anxiety for many families. Although different guidelines define RM differently—some classify it as two or more consecutive pregnancy failures, while others define it as three or more—all have a profoundly negative medical and psychological impact on families [1, 2]. In recent years, there has been significant progress in our knowledge of the pathogenesis of RM, with new insights, such as altered RNA processing and impaired ribosome function [3], decidual stromal cell (DSC) ferroptosis associated with abnormal iron metabolism [4], and the intricate interplay of intercellular communication involving macrophages at the maternal–fetal interface [5]. These discoveries are closely related to the underlying mechanisms that contribute to the development of RM, further enhancing our understanding of this complex condition. However, the detailed mechanisms of this condition at the molecular level remain unclear, especially given the advances in omics studies, which have revealed that the pathogenesis of RM is far more complex than currently understood [6].

The decidua functions as a biosensor for the embryo’s interaction with the mother, and its condition is directly related to embryo implantation and subsequent pregnancy [7]. The formation of the decidua, which comprises a heterogeneous population of cells, such as DSCs and decidual immune cells (DICs), is a critical event during the establishment of pregnancy. As the decidua can promote vascular remodeling and protect the embryo from maternal immune rejection [8], abnormalities in the decidua are closely associated with pathological pregnancies, including RM [9, 10]. For example, alterations in the decidual cytokine profile might trigger miscarriage by activating a variety of aberrant maternal-fetal signaling networks [11]. However, a full understanding of the molecular events in the decidua associated with RM remains a major challenge. Undoubtedly, identifying more decidual factors related to RM should accelerate the investigation of the pathogenesis of RM.

In the contemporary era, the field of bioinformatics has been undergoing rapid development, introducing a number of novel technological tools and facilitating significant breakthroughs in both basic research and clinical applications. Transcriptomics methodologies, including microarrays, bulk RNA sequencing (RNA-seq), and single-cell RNA-seq (scRNA-seq), allow the exploration of expression and regulation at the gene level [12]. Proteomics technologies, such as iTRAQ-tagged quantitative proteomics and protein microarrays, enable the study of proteins expressed in tissues or cells, both qualitatively and quantitatively [12]. Certainly, these bioinformatics tools have also been successfully applied to identify novel factors potentially involved in RM. However, the translation from mRNA to protein is complex and intricate [13], and to the best of our knowledge, most current studies on RM have focused on either the transcriptome or proteome independently, without a joint analysis of both [14–16].

Thus, to present novel ideas and clues for the investigation of RM pathogenesis and the development of future RM clinical treatments, this study was conducted to identify RM-related hub genes from decidual tissues of RM patients using combined transcriptomics and proteomics tools. We also aimed to identify upstream miRNAs of these hub genes and potential therapeutic drugs targeting these hub genes through systematic bioinformatics analysis. Then, the differential expression levels of these identified hub genes were validated in decidual tissues of RM patients, as well as in uterine tissues of LPS-induced early pregnancy loss (EPL) mice, using real-time quantitative PCR (RT-qPCR) and/or Western blot (WB) analyses. Additionally, we utilized single-cell sequencing technology to extend the investigation of hub gene expression into distinct cell types within the decidual tissues, facilitating the selection of suitable cell types for preliminary mechanistic explorations.

## Materials and methods

### Human decidual sample collection

Human decidual tissues from RM patients and normal control women (NC) who had no history of miscarriage and were undergoing legal, voluntary terminations of early pregnancy were collected from the Second Hospital of Tianjin Medical University between April and October 2017. The current miscarriage in RM patients was objectively confirmed by transvaginal ultrasound examination. All patients were excluded from classical risk factors, including abnormal parental karyotypes, uterine anatomical abnormalities, infectious diseases, luteal phase defects, diabetes mellitus, thyroid dysfunction, and hyperprolactinemia. The collected decidual tissues were immediately washed twice with sterile, glucose-free PBS solution to remove excess blood and stored at −80 ^∘^C. Decidual tissues were collected and used after obtaining informed consent from participants, with approval from the Second Affiliated Hospital of Tianjin Medical University (KY2017K002) and the Shanghai Institute for Biomedical and Pharmaceutical Technologies (PJ2018-06).

### Library construction for cDNA and illumina sequencing

Total RNA was extracted from the decidual tissues of three RM samples and three control samples using conventional experimental protocols. The extracted RNA then underwent rigorous quality control. The cDNA library was developed using the NEBNext^®^ Ultra™ RNA Library Prep Kit for Illumina^®^. Total mRNA was enriched with Oligo(dT) magnetic beads to obtain purified mRNA, which was then fragmented into short fragments to synthesize cDNA. The double-stranded cDNA was end-repaired, had A-tails added, and was ligated to sequencing adaptors. cDNAs of 250–300 bp were selected for PCR amplification, and the PCR products were further purified to obtain the library. The libraries were quantified using a Qubit 2.0 Fluorometer and qRT-PCR, and sequenced on the Illumina platform.

### Read quality control and data processing of transcriptome

Quality control of the acquired sequencing data was performed, including removing reads containing adapters, reads with unidentifiable base information, and low-quality reads, to obtain clean reads for subsequent analyses. The HISAT2 software was used to quickly and accurately map the positional information of clean reads to the reference genome. The featureCounts tool in the Subread software was applied to count the number of reads covered by each gene from the beginning to the end of the genome, filtering out reads containing more than 10% low-quality bases, those that could not be matched in pairs, and those matching multiple regions of the genome. Gene expression levels were quantified individually for decidual tissue samples from RM patients and control (NC) women, then combined to obtain expression matrices for all six samples.

### Identification of DEGs

The DESeq2 package in R (version 4.2.2) was used to screen differentially expressed genes (DEGs) between RM and control groups. An absolute value of log2FoldChange >1 and *P* value < 0.05 were used to determine DEGs. The ggplot2 package and Complex Heatmap package were used to construct volcano plots and heat maps to visualize the distribution and clustering of DEGs, respectively.

### Enrichment analysis of DEGs

To better understand the function of DEGs, all DEGs identified between the RM and Control groups were imported into the DAVID (https://david.ncifcrf.gov) database for enrichment analysis. All visualizations were created using an online mapping platform (http://www.bioinformatics.com.cn). *P* < 0.05 as a cutoff point to determine if the pathway or Go term is significantly enriched or not.

### GSEA analysis in transcriptome

Gene set enrichment analysis (GSEA) provides a more thorough understanding of potential regulatory mechanisms and functions, considering genes that may not differ significantly individually but could play key roles in the overall biological process. GSEA was performed using the GSEABase, clusterProfiler, enrichplot, and GOplot packages, along with the gseKEGG, gseaplot2, and gseGO functions, to identify KEGG pathways and Gene Ontology (GO) items. Notably enriched gene sets were identified as those with a *P* value <0.05.

### Protein extraction, SDS-PAGE, and filter-aided sample preparation (FASP)

Decidual tissue from RM patients and control (NC) women was added to an appropriate volume of SDT lysis buffer (4% SDS, 100-mM Tris-HCl, pH 7.6). MP Fastprep-24 was used to homogenize the tissue, followed by ultrasound to ensure adequate lysis. The lysate was placed in a boiling water bath for 15 min and then centrifuged at 14,000 *g* for 15 min. The supernatant obtained after centrifugation was filtered through a 0.22-µm membrane and then transferred to a new centrifuge tube. After quantifying the protein concentration using the BCA kit, samples were aliquoted and stored at −80 ^∘^C. We used 20 µg of each sample to examine and evaluate whether the quality of the samples met the experimental requirements using 12% SDS-PAGE (constant voltage of 250 V for 40 min). Next, 30 µL of the tissue protein solution was digested.

### iTRAQ labeling, high PH RP fractionation and mass spectrometry analysis

Peptides (100 µg) from each sample were labeled using the iTRAQ Reagent-4/8plex Multiplex Kit, following the manufacturer’s instructions. The labeled peptides were mixed and then classified using the Agilent 1260 Infinity II HPLC. The C18 column was equilibrated first, and then the samples were loaded onto the column for separation. Absorbance at 214 nm was monitored during the separation and elution process, and the eluted fractions were collected, lyophilized, re-dissolved, and combined. Each sample was separated using the Easy nLC system. Next, the analytical column was equilibrated, and the samples were loaded onto it for separation. The separated samples were analyzed using a Q-Exactive Plus mass spectrometer.

### Data processing of proteome

The MASCOT 2.5 server and Proteome Discoverer 2.1 software were used to process the raw data obtained from the mass spectrometer. A false-positive rate (FPR) <0.01 was used as a screening criterion, and the protein sequence database (Uniprot_HomoSapiens_159615_20170811.fasta) was selected to obtain dependable qualitative results and reliable quantitative information on protein abundance.

### Identification of DEPs

Identification of differentially expressed proteins (DEPs) between RM patients and Control women was performed using the limma package in R 4.2.2. DEPs were screened based on statistical significance (*P* value < 0.05) and fold change (≤ 0.83 for downregulation in the RM group or ≥ 1.2 for upregulation in the RM group). A volcano plot was created using the ggplot2 package, with upregulated proteins labeled in red and downregulated proteins shown in blue. Clustering heatmap analysis of DEPs was performed using the ComplexHeatmap package.

### Enrichment analysis of DEPs

To better understand the function of DEPs, all DEPs identified between the RM and Control groups were imported into the DAVID (https://david.ncifcrf.gov) database for enrichment analysis. All visualizations were created using an online mapping platform (http://www.bioinformatics.com.cn). *P* < 0.05 was used as the cutoff point to determine if a pathway or GO term was significantly enriched.

### GSEA analysis in proteome

The method for conducting GSEA analysis based on KEGG pathways and GO annotation in the proteome was the same as that used for GSEA analysis in the transcriptome. A ridge plot was used to display the expression and distribution of all significantly enriched pathways and their core enriched proteins, and a bubble diagram generated using the ggplot2 package was employed to illustrate the top ten significantly enriched GO items.

###  PPI analysis

The study of protein–protein interactions (PPI) is crucial for revealing the pathological mechanisms of many diseases and understanding the complex molecular relationships within organisms. To investigate the interactions between the DEPs of the RM group and the control group, the DEPs were input into the STRING 12.0 database (https://cn.string-db.org/) to obtain the PPI network, which was then imported into Cytoscape 3.9.1 software for further analysis. CytoHubba, a plugin in Cytoscape, provides 12 topological algorithms, and we selected the maximum clique centrality (MCC) algorithm to identify the top ten hub proteins. MCODE, another plugin of Cytoscape, was used to calculate and extract significant protein interaction modules. After performing MCODE analysis, we extracted the nodes from the two best-scoring modules as core hub proteins.

### Identification of RM-related hub genes

The association between genes and proteins was displayed using a nine-quadrant graph in R 4.2.2. The online tool jvenn (https://jvenn.toulouse.inra.fr/app/index.html) was used to create a Venn diagram by taking the intersection of three components, which included shared DEGs (proteins), hub proteins (genes) obtained using the MCC algorithm, and hub proteins (genes) identified using MCODE plugins.

### Identification of transcription factors (TFs) and MicroRNAs

NetworkAnalyst 3.0 (https://www.networkanalyst.ca) is an online platform that can identify TFs. We selected the JASPAR database (one of the four databases) available on NetworkAnalyst 3.0 to construct and visualize the TF-gene network. The miRNet (https://www.mirnet.ca/) database integrates information from 14 microRNA databases, including miRanda and starBase. We used it to predict and visualize the miRNAs that target hub genes (proteins).

### ScRNA-seq data download and processing

To understand the distribution and differential expression of RM-related hub genes in different cell types within the decidual tissue of RM patients and NC women, we analyzed the single-cell dataset GSE214607, downloaded from the GEO (https://www.ncbi.nlm.nih.gov/geo/) database. The GSE214607 dataset, comprising five normal women (NC) and three RM patients, was sequenced using the 10× Genomics platform. We used the Seurat package in R 4.2.2 for subsequent analysis. The PercentageFeatureSet function in the Seurat package was applied to calculate the proportion of mitochondria in each cell for quality control. Low-quality cells, defined as cells with fewer than 200 genes, more than 6000 genes, or over 10% mitochondrial content, were excluded from our analysis. Normalization and identification of highly variable genes were performed using the NormalizeData and FindVariableFeatures functions of the Seurat package. The ScaleData function was used to scale all genes, and the Harmony package was employed to remove batch effects between samples from RM patients and NC women. Dimensionality reduction and clustering analysis were performed, and marker genes of all cell populations were extracted using the FindAllMarkers function. Cell populations were annotated by gathering marker genes for each cell type through literature searches after automated annotation using the SingleR package, followed by manual correction of the automated results. Finally, using the FindMarkers function, we investigated the differential expression of hub genes in different cell types of RM patients compared to NC women. The markers for different cells are as follows: NK cells (FGFBP2, PRF1, NCAM1); DSC cells (YBX3, VCL, ZBTB20); macrophages (CD163, CD14, MSR1); glandular epithelial cells (LINC01502, CXCL14, GPX3); EVT (FN1, PAPPA2); T cells (CD3D, CD3G, CD8A); cytotrophoblast cells (PAGE4, PEG10); B cells (CD79A, MS4A1, IGHM); and neutrophils (TPSB2, TPSAB1).

### Drug prediction

The DSigDB database, built on the Enrichr platform (http://amp.pharm.mssm.edu/Enrichr), was used to identify potential drugs that target RM-related hub genes. The top ten candidate drugs were determined using *P* value. The combined score can be used to assess the association of a drug to a gene if the *P* value is significant.

### Real-time quantitative PCR (RT-qPCR)

We selected nine decidual tissue samples from RM patients and nine from control subjects to validate the expression levels of five hub genes using qPCR detection. Total RNA was extracted using Trizol reagent (Invitrogen, USA). The concentration of the extracted RNA was measured using the Infinite^®^ 200 PRO (TECAN, Switzerland). PrimeScript RT Master Mix (TAKARA, Japan) was used for cDNA synthesis. According to the instructions of the TB Green^®^ Premix Ex Taq™ qPCR kit, the cDNA solution obtained by reverse transcription was diluted threefold and used as a template for amplification using the LightCycler 480 II Real-Time Fluorescence Quantitative PCR System (Roche, Switzerland) with specific primers. The sequences of specific primers are listed in Table S1. MicroRNA detection utilized the ReverTra Ace-α-TM FSK101 kit (TOYOBO, Japan) and the SYBR Green Premix Pro Taq HS qPCR Kit (Accurate Biology, China). The relative expression levels of hub genes and microRNAs were calculated using the 2^−ΔΔCt^ method.

### Establishment of the LPS-induced EPL mouse model

Adult male and female C57BL/6 mice (eight weeks old) were purchased from SIPPR/BK Laboratory Animal Company (Shanghai, China). All experiments were conducted in accordance with standard laboratory animal care protocols approved by the Institutional Animal Care Committee of the Shanghai Institute for Biomedical and Pharmaceutical Technologies (#2022-52). The LPS-induced abortion model was established according to previous reports with slight modifications [17, 18]. Briefly, female mice were naturally mated with males in a 2:1 ratio. Mice were inspected every morning for vaginal plugs, and the day of detection was designated as GD 0.5. Pregnant females were intraperitoneally injected with 0.25 mg/kg LPS (Sigma-Aldrich, St. Louis, MO, USA) at GD 6.5 to induce abortion (LPS-treated group). The control group received an equal volume of sterile saline solution. All mice were sacrificed at GD 7.5 (24 h after LPS treatment).

### Protein extraction and Western blotting

The method of protein extraction and Western blotting was described in our previous study [19]. Briefly, proteins were extracted using RIPA lysis buffer (Sangon Biotech), supplemented with 1-mM PMSF and a protease inhibitor cocktail (Selleck, Shanghai, China), and then treated with an ultrasonic cell crusher. Following this, the mixture was centrifuged at 12,000 *g* for 15 min at 4 ^∘^C. After centrifugation, the supernatant was transferred to a new 1.5-mL EP tube, and the total protein concentration was determined using the Bradford method. Next, 5× loading buffer was added to the supernatant, which was then boiled in a metal bath at 100 ^∘^C for 7 min. Approximately 30 µg of protein per lane was separated using sodium dodecyl sulfate-polyacrylamide gel electrophoresis (SDS-PAGE) and then transferred to nitrocellulose membranes (Merck Millipore, Darmstadt, Germany). The membranes were blocked with 5% defatted milk powder for 1 h and then washed using TBST. Following this, the membranes were incubated with primary antibodies at 4 ^∘^C overnight. The primary antibodies included anti-actin (1:2000, 66009-1-Ig, Proteintech) and anti-ISG15 (1:2000, 15981-1-AP, Proteintech). The membranes were then washed with TBST and incubated with corresponding secondary antibodies for 1 h. Finally, visualization was performed using the Odyssey CLx Imaging System (LI-COR, USA).

### T-HESCs culture and decidualization treatment

T-HESC, an immortalized human endometrial stromal cell (hESC) line, was kindly provided by Professor Haibin Wang, School of Medicine, Xiamen University, China. The medium components included phenol red-free DMEM/F-12 (Gibco), 10% charcoal-stripped fetal bovine serum (CS-FBS) (Biological Industries, Cromwell, CT), 1-mM sodium pyruvate (Sigma), 1% insulin-transferrin-selenium (ITS, Gibco), and 500 ng/mL puromycin (Sigma). T-HESC cells that reached 70% confluence were seeded into six-well plates at a density of 3 × 10^5^ cells per dish for 24 h, and then *in vitro* decidualization was initiated. Reagents used for *in vitro* decidualization included phenol red-free DMEM/F-12, 2% CS-FBS, 10-nM β-estradiol (E2, Sigma), 1-µM medroxyprogesterone 17-acetate (MPA, Sigma), and 0.5-mM cyclic adenosine monophosphate (cAMP, Sigma). These stimuli were replaced every two days. Finally, the cells were collected at different time points.

### Ethical statement

The study was approved by the Second Affiliated Hospital of Tianjin Medical University (Approval Number: KY2017K002) and the Shanghai Institute for Biomedical and Pharmaceutical Technologies (Approval Number: PJ2018-06).

### Statistical analysis

Baseline information was tested using SPSS (version 20) software. A two-tailed unpaired Student’s *t*-test was used to calculate the *P* value for age, gestational weeks, and gravidity, while the Mann–Whitney *U* test was used to calculate the *P* value for parity and abortion times. GraphPad Prism 8.0 was used to analyze qPCR results using a *t*-test. A *P* < 0.05 was considered statistically significant.

## Results

### Baseline characteristics of recruited human subjects

The same batch of decidual tissue samples collected from three RM patients and three NC women was used for the transcriptomic and proteomic analyses. There was no significant difference in age, gestational weeks, gravidity, or parity between the RM group and the NC group, whereas the number of abortions was significantly different between the two groups (*P* < 0.05) (Table S2). Table S3 showed the characteristics of the RM patients and NC women who were recruited during the early stages of pregnancy in order to validate the findings of the bioinformatics analysis.

### Transcriptomic analysis of RM

**Figure 1.**
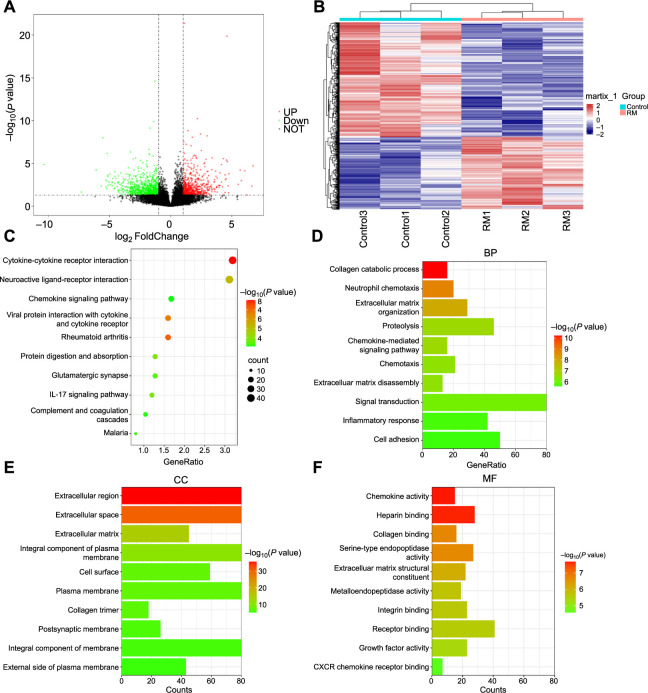
**DEGs in decidual tissues of RM patients screened through RNA-seq analysis.** (A) The volcano plot of DEGs between RM patients (RM, *n* ═ 3) and normal early pregnant women (Control, *n* ═ 3). Red dots represent upregulated genes in the RM group, green dots represent downregulated genes, and black dots represent genes with no significant differences between the RM and Control groups. (B) Heatmap clustering of DEGs between the RM and Control groups. Red indicates upregulation in the corresponding group, while blue indicates downregulation. RM: Decidual tissues of RM patients; Control: Decidual tissues of normal early pregnant women. (C) Top ten pathways associated with DEGs enriched by KEGG analysis. (D) Top ten BP associated with DEGs enriched by GO analysis. (E) Top ten CC associated with DEGs enriched by GO analysis. (F) Top ten MF associated with DEGs enriched by GO analysis. DEG: Differentially expressed gene; BP: Biological process; CC: Cellular component; MF: Molecular function; RM: Recurrent miscarriage; GO: Gene ontology; RNA-seq: RNA sequencing.

A total of 1823 DEGs were identified between the decidual tissues of the RM group and the Control group through transcriptome analysis, including 706 upregulated genes and 1117 downregulated genes in the RM group based on the log2FC and *P* value criteria used. DEGs are represented by red and green points in the volcano plot (Figure 1A). The expression levels of all screened DEGs were clustered in a heatmap (Figure 1B). Our results showed significant intergroup differences between the two groups, with high similarity of samples within each group, indicating effective clustering and good reproducibility of our samples.

The functions of the DEGs were explored through KEGG and GO analyses using the DAVID database. The top ten KEGG results revealed that DEGs were primarily enriched in inflammatory response pathways, including cytokine–cytokine receptor interaction, rheumatoid arthritis, and the IL-17 signaling pathway, among others (Figure 1C). GO annotation includes biological process (BP), cellular component (CC), and molecular function (MF) items. As illustrated in Figure 1D–1F, the top ten items in BP, CC, and MF were all related to the extracellular matrix (ECM), and BP and MF were both linked to inflammation.

**Figure S1.**
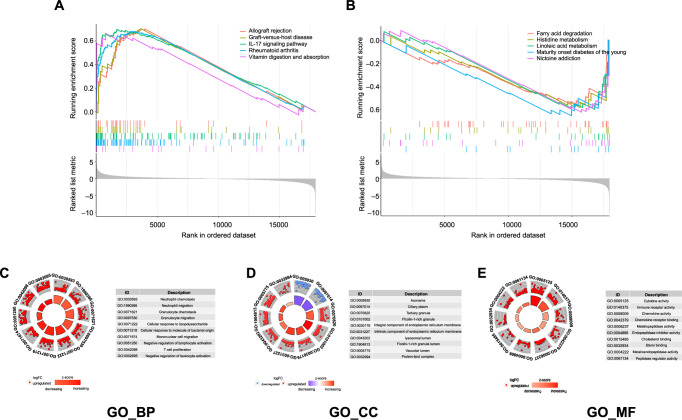
(A) Represents the top five upregulated pathways after GSEA analysis; (B) Represents the top five downregulated pathways after GSEA analysis; (C) Represents the top ten BP items after GSEA analysis; (D) Represents the top ten CC items after GSEA analysis; represents the top ten MF items after GSEA analysis. MF: Molecular function; GSEA: Gene set enrichment analysis; CC: Cellular component; BP: Biological process.

GSEA analysis was performed to identify potential enrichment items among non-DEGs and validate the results of DEGs [20]. After GSEA analysis, the significantly enriched signaling pathways and GO items were identified using a *P* value threshold of < 0.05. Based on the enrichment score, the top five upregulated pathways associated with RM patients included Allograft rejection, Graft-versus-host disease, IL-17 signaling pathway, Rheumatoid arthritis, and Vitamin digestion and absorption (Figure S1A). The top five downregulated pathways associated with RM patients included Fatty acid degradation, Histidine metabolism, Linoleic acid metabolism, Maturity-onset diabetes of the young, and Nicotine addiction (Figure S1B). Notably, the top-ranked pathways primarily play roles in inflammation, immunity, and metabolic functions. The significantly enriched GO annotations are shown in Figure S1C–S1E; these items are predominantly related to immune regulation, catalytic activity, and metabolic processes, with the most enriched GO items associated with upregulated genes in the decidual tissues of RM patients.

### Proteomic analysis of RM

**Figure 2.**
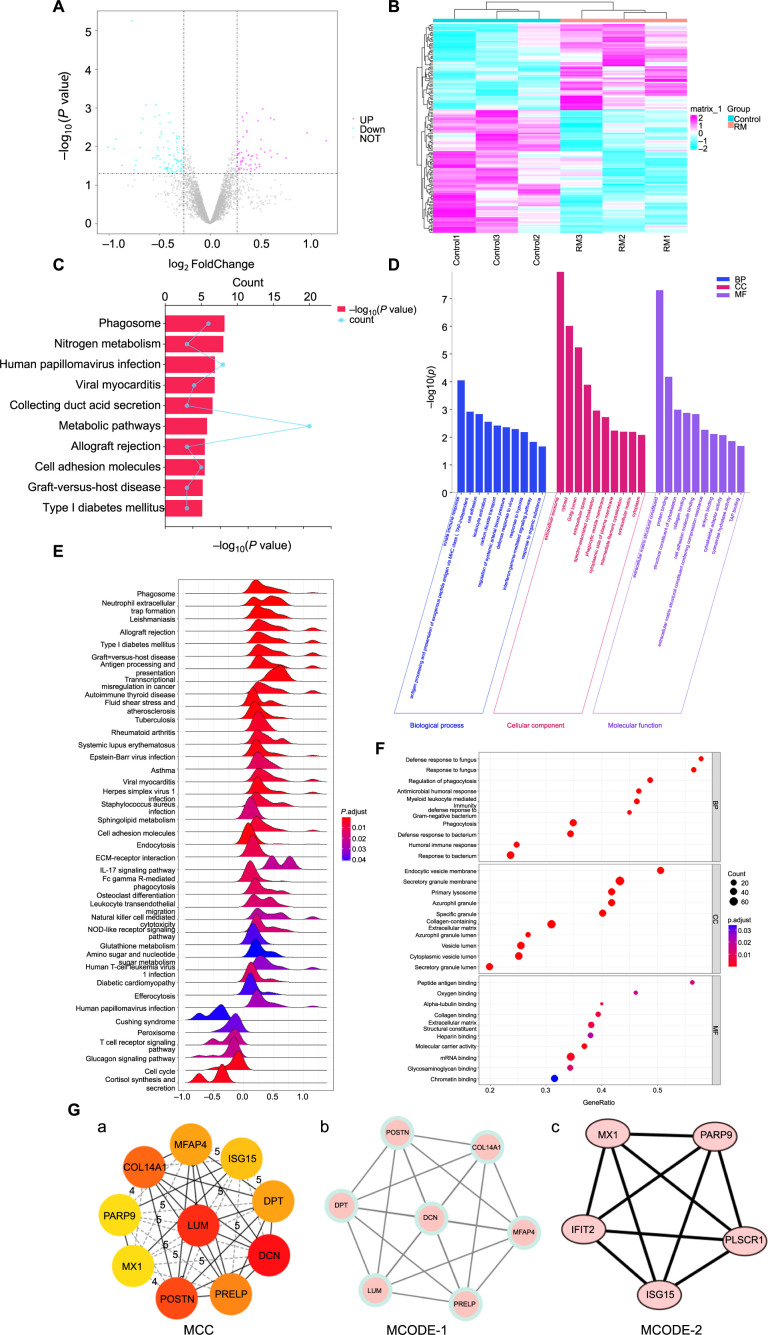
**DEPs in decidual tissues of RM patients identified through iTRAQ analysis.** (A) The volcano plot of DEPs between RM patients (RM group, *n* ═ 3) and normal early pregnant women (Control group, *n* ═ 3). Red dots represent upregulated proteins in the RM group, blue dots represent downregulated proteins, and gray dots represent proteins with no significant differences between the RM and Control groups. (B) Heatmap clustering of DEPs between the RM and Control groups. Red indicates upregulation in the corresponding group, while blue indicates downregulation. RM: Decidual tissues of RM patients (*n* ═ 3); Control: Decidual tissues of normal early pregnant women (*n* ═ 3). (C) Top ten pathways associated with DEPs enriched by KEGG analysis. (D) Top ten BP, CC, and MF associated with DEPs. (E) All pathways associated with DEPs enriched by GSEA analysis. (F) Top ten BP, CC, and MF items associated with DEPs enriched by GSEA analysis. (G) (a) represents the top ten hub proteins identified using the MCC algorithm; (b) and (c) represent 12 hub proteins identified using the two highest-scoring modules obtained with the MCODE plugin. BP: Biological process; CC: Cellular component; MF: Molecular function; RM: Recurrent miscarriage; GSEA: Gene set enrichment analysis; DEP: Differentially expressed protein; MCC: Maximum clique centrality.

According to our filter criteria, we identified 148 DEPs from the proteome analysis, including 61 upregulated proteins and 87 downregulated proteins in the RM group. DEPs are represented by red and blue points in the volcano plot (Figure 2A). The expression levels of all identified DEPs were clustered in a heatmap (Figure 2B). The heatmap demonstrates the similarity of samples within each group and the differences between the two groups, indicating that our samples are suitable for subsequent analysis.

To better understand the function of DEPs, we performed KEGG and GO analyses. The top ten KEGG pathways included Phagosome, Nitrogen metabolism, Metabolic pathways, Allograft rejection, Cell adhesion molecules, Graft-versus-host disease, Type I diabetes mellitus, and others (Figure 2C). These significantly enriched KEGG pathways are mainly related to inflammation and metabolism, which aligns with the results from the transcriptome analysis. The results of the GO (BP, CC, and MF) items also indicated connections to inflammation and ECM (Figure 2D).

GSEA analysis was conducted to identify potential enrichment items among non-DEPs and validate the results of DEPs [20]. All pathways enriched after GSEA analysis are presented in a ridge plot, which includes 34 upregulated pathways and six downregulated pathways (Figure 2E). Based on the enrichment score, the top ten upregulated pathways associated with RM patients included Allograft rejection, Asthma, Autoimmune thyroid disease, Rheumatoid arthritis, Graft-versus-host disease, and Type I diabetes mellitus. The top five downregulated pathways associated with RM patients included Cell cycle, Cortisol synthesis and secretion, Cushing syndrome, Glucagon signaling pathway, and Peroxisome. The results are highly consistent with RNA-seq analysis, primarily highlighting contributions to immunity and metabolism. We also visualized the top ten enriched items of CC, BP, and MF using a bubble diagram (Figure 2F), which were mainly associated with immune function and cell migration activity.

The 148 DEPs were uploaded to the STRING database for PPI analysis. Two plug-ins, CytoHubba and MCODE in Cytoscape, were used to identify key hub proteins based on the PPI network. The top ten key hub proteins, including LUM, DCN, DPT, ISG15, MFAP4, COL14A1, PARP9, MX1, POSTN, and PRELP, were calculated using the MCC algorithm of CytoHubba (Figure 2G-(a)). Modular analysis of the PPI network using the MCODE plugin identified two modules with the highest scores, with LUM, DCN, DPT, ISG15, MFAP4, COL14A1, PARP9, MX1, POSTN, PRELP, IFIT2, and PLSCR1 identified as key hub proteins (Figure 2G-(b) and (c)), encompassing all ten key hub proteins retrieved from the MCC algorithm. The results obtained through both methods were highly reproducible.

### Combined analyses of RNA-seq and iTRAQ for RM

**Figure 3.**
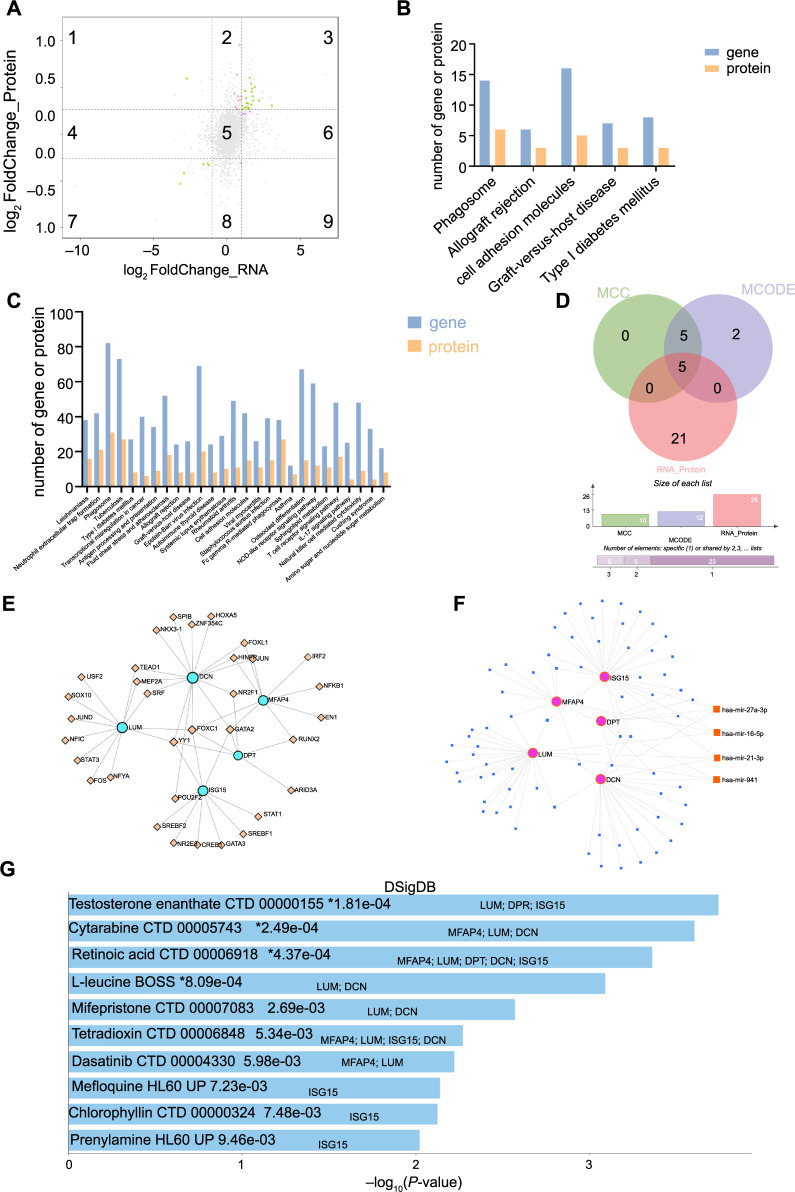
**Identification of RM-related hub genes through integrative analyses of DEGs and DEPs in decidual tissues of RM patients.** (A) Nine-quadrant graph of correlation analysis from the transcriptomic and proteomic data of decidual tissues of RM patients and normal early pregnancies; (B) Top five pathways associated with both DEGs and DEPs in the decidual tissues of RM patients enriched by KEGG analysis; (C) Pathways associated with both DEGs and DEPs in the decidual tissues of RM patients enriched by GSEA analysis; (D) Venn diagram showing the identification of RM-related hub genes. MCC: Top 10 hub proteins identified from DEGs and DEPs using the MCC algorithm; MCODE: Top 12 hub proteins identified using the MCODE plugin; RNA_Protein: A total of 26 proteins common to both DEGs and DEPs; (E) TF network associated with the RM-related five hub genes using the JASPAR database; (F) Interaction network diagram of potential upstream microRNAs of the RM-related five hub genes using the miRNet database; (G) Top ten proposed drugs targeting RM-related hub genes identified using the DsigDB database. RM: Recurrent miscarriage; GSEA: Gene set enrichment analysis; DEP: Differentially expressed protein; DEG: Differentially expressed gene; TF: Transcription factor; MCC: Maximum clique centrality.

A nine-quadrant graph was created by integrating both gene and protein analyses (Figure 3A). The results showed that most genes or proteins detected by the transcriptome and proteome exhibited no significant difference between the RM and Control groups (quadrant 5). Quadrants 3 and 7 display significant differences and the same trend in 26 genes or proteins (green dots), of which 21 genes (proteins) are significantly upregulated and five genes (proteins) are significantly downregulated. Only one gene (protein) showed significant differences with opposite trends (quadrant 1). The pink and purple dots in the remaining quadrants represent DEGs or DEPs detected by the transcriptome or proteome alone. After a joint analysis, five common KEGG pathways based on DEGs and DEPs were identified (Figure 3B), primarily related to inflammation and metabolism. Additionally, 27 common pathways were found through GSEA analysis between the transcriptome and proteome (Figure 3C), which are also mainly concentrated on inflammation, infection, and metabolic functions.

To identify RM-related hub genes (proteins), ten key proteins obtained from the MCC algorithm, 12 hub proteins identified through the MCODE plugin, and the 26 genes (proteins) shared between DEGs and DEPs were analyzed using a Venn diagram (Figure 3D). This analysis yielded five RM-related hub genes (proteins): DCN, LUM, ISG15, MFAP4, and DPT, all of which were notably upregulated in the decidual tissue of RM patients.

Transcription factors (TFs) play a crucial role in regulating gene transcription and influencing diseases [21]. Therefore, we further screened TFs related to hub genes. The TF-gene network, consisting of 38 nodes and 52 edges, was constructed using NetworkAnalyst 3.0. A total of 33 TFs were identified (Figure 3E). Notably, FOXC1, located at the center of this network, can regulate all hub genes.

MicroRNAs have been confirmed to play important roles in the development, progression, and treatment of many diseases [22–24]. Thus, we predicted microRNAs associated with RM-related hub genes. We imported five hub genes into the miRNet database, which generated 75 miRNAs predicted to target our hub genes. Among all predicted miRNAs, hsa-mir-27a-3p, hsa-mir-16-5p, hsa-mir-21-3p, and hsa-mir-941 are relevant to three hub genes (Figure 3F).

To explore potential drugs for RM therapy, we used the DSigDB database to predict possible drugs targeting the five hub genes. The top ten candidate agents and their corresponding target genes are illustrated in Figure 3G. Detailed information can be found in Table S4.

### Single-Cell RNA-seq analysis of RM-related hub genes expression levels

**Figure 4.**
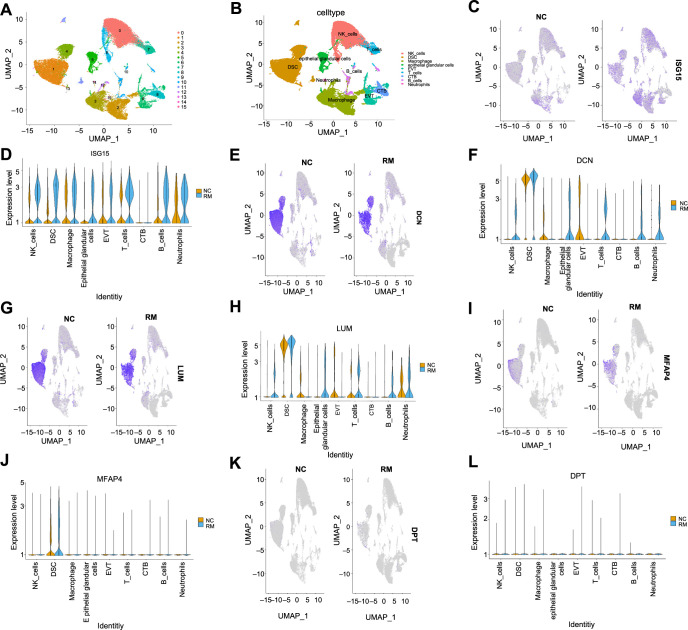
**Expression patterns of RM-related hub genes in various cell populations between RM patients and normal early pregnant women using the single-cell dataset GSE214607.** (A) UMAP visualization of 16 cell clusters contained in human decidual tissues during early pregnancy; (B) UMAP plot of nine cell types contained in human decidual tissues during early pregnancy; (C/E/G/I/K) Expression profiles of *ISG15/DCN/LUM/MFAP4/DPT* in different cell types of human decidua during early pregnancy displayed by UMAP plot; (D/F/H/J/L) Expression profiles of *ISG15*/*DCN*/*LUM*/*MFAP4*/*DPT* in different cell types displayed by violin plot. DSC: Decidual stromal cell; EVT: Extravillous trophoblast; CTB: Cytotrophoblast; RM: Recurrent miscarriage patients (*n* ═ 3); NC: Normal early pregnant women (*n* ═ 5).

To analyze and compare the expression levels of five hub genes across various cell types in the decidual tissues of RM patients and control women, a single-cell RNA sequencing dataset containing three decidual tissue samples from RM patients and five for further analysis. After quality control, the RM group included 25,188 cells and 24,849 genes, while the control group comprised 36,302 cells and 25,002 genes, which were used for subsequent analysis. Sixteen cell populations were identified through dimensionality reduction and clustering (Figure 4A), and these were subsequently annotated into nine cell types (Figure 4B), including macrophages, NK cells, EVT cells, DSC cells, and others that are predominant at the maternal–fetal interface. The expression status of the five hub genes in various cell types of decidual tissue was observed using UMAP plots and violin plots. As shown in Figure 4C–4L, compared to control women, the expression levels of the five hub genes were all higher in the DSC cells of RM patients. Among them, ISG15 was abundant in all cell types and showed upregulation in the RM group, whereas DPT expression was consistently lower across all cell types. The FindMarkers function, along with the Wilcoxon rank-sum test, was used to determine whether the expression levels of the hub genes differed significantly in DSC cells between the two groups, and the results showed that the expression of all five hub genes was significantly upregulated in DSC cells of the RM group (*P*_val< 0.05, avg_log2FC > 0.3).

**Figure 5.**
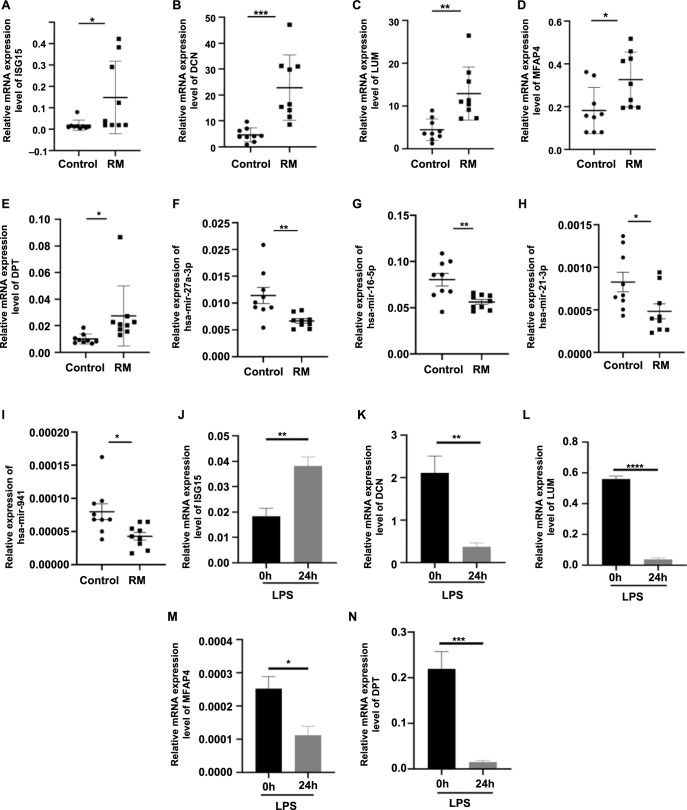
**Expression levels of five hub genes and four upstream miRNAs in human decidual tissues and mouse uterine tissues detected by qPCR analysis.** (A–E) The relative expression levels of *ISG15*, *DCN*, *LUM*, *MFAP4*, and *DPT* in human decidual tissues during early pregnancy; (F–I) The relative expression levels of miR-27a-3p, miR-16-5p, miR-21-3p, and miR-941 in human decidual tissues during early pregnancy; (J–N) The relative expression levels of *Isg15*, *Dcn*, *Lum*, *Mfap4*, and *Dpt* in mouse uterine tissues at day 7.5 of pregnancy (vaginal plug ═ day 0.5 of pregnancy). RM: Recurrent miscarriage patients (*n* ═ 9); Control: Normal early pregnant women (*n* ═ 9); 0 h: Day 7.5 pregnant mice without LPS treatment (*n* ═ 4); 24 h: Day 7.5 pregnant mice treated with LPS (0.25 mg/kg) at day 6.5 of pregnancy (*n* ═ 3); *P < 0.05, **P < 0.01, ***P < 0.001, ****P < 0.0001. qPCR: Quantitative PCR.

**Figure 6.**
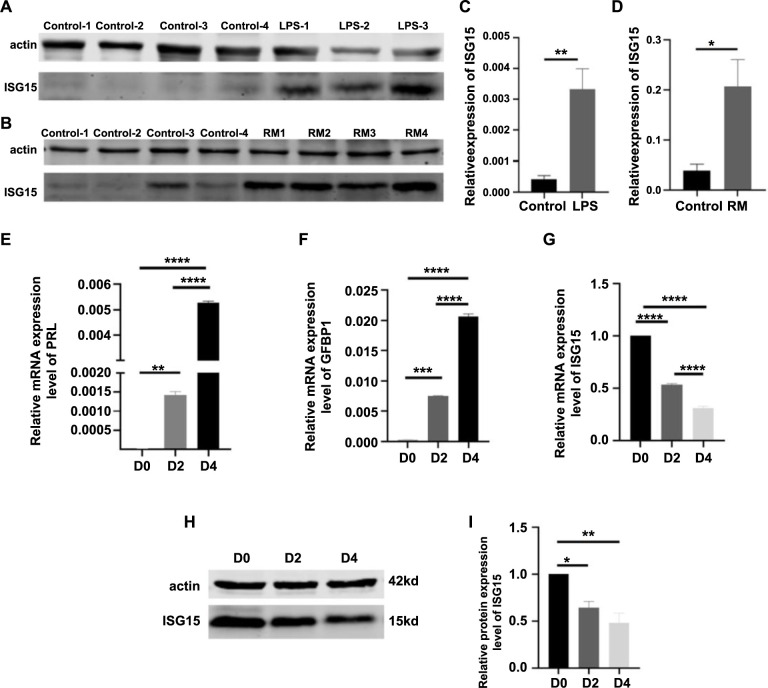
**Expression levels of ISG15 in human decidual tissues, mouse uterine tissues, and T-HESC cells as analyzed by Western blot and RT-qPCR.** (A) A representative Western blot image showing ISG15 protein levels in mouse uterine tissues. Control: Mice at day 7.5 of pregnancy without LPS treatment; LPS 1-3: Mice at day 7.5 of pregnancy treated with LPS (0.25 mg/kg) at day 6.5 of pregnancy. (B) A representative Western blot image showing ISG15 protein levels in human decidual tissues during early pregnancy. Control 1-4: Normal early pregnant women; RM 1-4: RM patients. (C) Relative uterine expression level of ISG15 protein in mice. Control: Mice at day 7.5 of pregnancy without LPS treatment (*n* ═ 4); LPS: Mice at day 7.5 of pregnancy treated with LPS (0.25 mg/kg) at day 6.5 of pregnancy (*n* ═ 3). (D) Relative expression level of ISG15 protein in human decidual tissues. Control: Normal early pregnant women (*n* ═ 9); RM: Recurrent miscarriage patients (*n* ═ 9). (E/G/F) Expression levels of PRL/*ISG15*/IGFBP1 mRNAs in T-HESCs. (H) A representative Western blot image showing ISG15 protein levels in T-HESCs. (I) Relative expression level of ISG15 protein during *in vitro* decidualization of T-HESCs. D0: Non-induced decidualization; D2: 48 h after induced decidualization with E2, MPA, and cAMP; D4: 96 h after induced decidualization with E2, MPA, and cAMP; *P < 0.05, **P < 0.01, ***P < 0.001, ****P < 0.0001; Cell experiments were independently repeated at least three times in triplicate (*n* ═ 3×2). RT-qPCR: Real-time quantitative PCR.

### Expression levels of five hub genes and four MicroRNAs in human decidual tissues of RM patients and NC women

To preliminarily validate the reliability of our combined transcriptome and proteome analysis, qPCR was used to detect and confirm the expression levels of five RM-related hub genes in decidual samples, including nine RM patients and nine control women. Compared with the control group, the results showed that *ISG15, LUM, DPT, DCN*, and *MFAP4* were significantly up-regulated in the decidual tissues of RM patients (Figure 5A–5E). Additionally, to assess whether hsa-mir-27a-3p, hsa-mir-16-5p, hsa-mir-21-3p, and hsa-mir-941 may influence RM through these hub genes, their expression was also examined in nine control decidual samples and nine RM samples. Their expression was found to be significantly down-regulated in RM patients (Figure 5F–5I). The hub gene expression levels were further confirmed using the LPS-induced mouse abortion model. The results showed that only the expression level of *ISG15* was consistent with our analysis results (Figure 5J–5N).

### ISG15 expression was increased in both uterine tissues of LPS-induced EPL mice and decidual tissues of RM patients but decreased during the *in vitro* decidualization of T-HESCs

We selected *ISG15*, a hub gene that showed similar trends in qPCR outcomes in the mouse model of miscarriage and in patients with RM, for further validation at the protein level. As illustrated in Figure 6A–6D, compared to control samples, LPS-induced mice exhibited higher levels of ISG15 protein expression. Similarly, decidual tissues from RM patients displayed elevated ISG15 protein expression compared to normal samples. A comprehensive analysis of single-cell sequencing data revealed that the five hub genes were significantly overexpressed in DSCs. Based on this finding, hESCs were selected for further study, and preliminary functional exploration was conducted.

**Figure S2.**
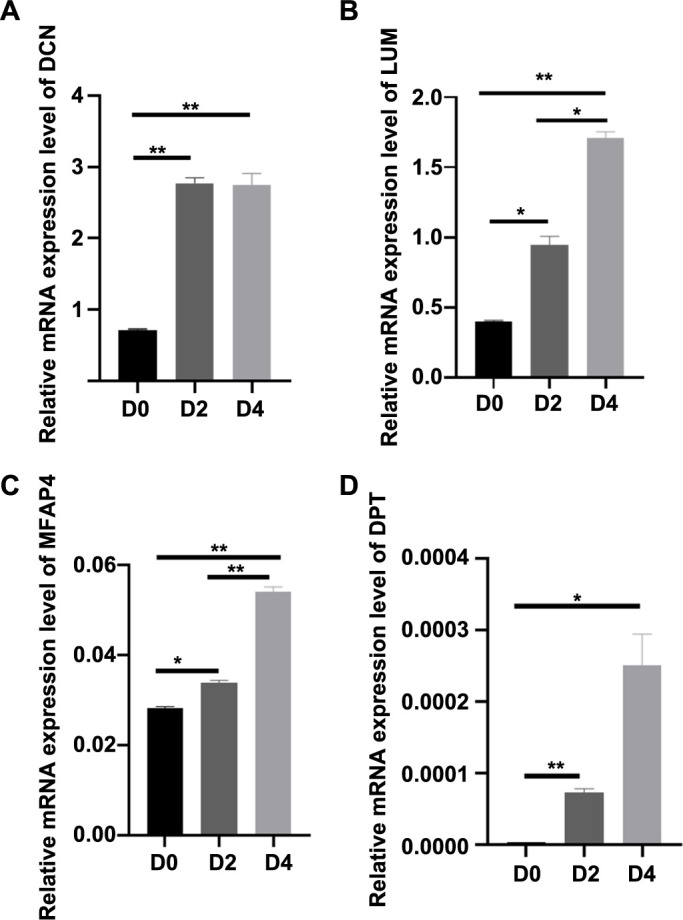
(A) Represents the relative expression level of *DCN* during *in vitro* decidualization of T-HESCs; (B) Represents the relative expression level of *LUM* during *in vitro* decidualization of T-HESCs; (C) Represents the relative expression level of *MFAP4* during *in vitro* decidualization of T-HESCs; (D) Represents the relative expression level of *DPT* during *in vitro* decidualization of T-HESCs. *P < 0.05, **P < 0.01; Cell experiments were independently repeated at least three times in triplicate (*n* ═ 3×2). D0: Non-induced decidualization; D2: 48 h after induced decidualization with a combined treatment of E2, MPA, and cAMP; D4: 96 h after induced decidualization with a combined treatment of E2, MPA, and cAMP; *DCN*: Decorin; *LUM*: Lumican; *MFAP4*: Microfibrillar-associated protein 4; *DPT*: Dermatopontin.

Given the importance of endometrial decidualization during pregnancy, the decidualization function was specifically tested. First, the expression levels of the decidual markers *PRL* and *IGFBP1* were assessed, both of which were found to be elevated (Figure 6A and 6F), indicating successful construction of the decidualization process. Additionally, our study revealed a significant decrease in the expression of *ISG15* during the decidualization process, both at the mRNA and protein levels (Figure 6G–6I). In contrast, RT-qPCR analysis showed that the expression levels of *MFAP4*, *LUM*, *DPT*, and *DCN* in T-HESCs were all significantly upregulated after *in vitro* decidualization (Figure S2), suggesting that they might play different roles in decidualization compared to ISG15.

## Discussion

This study identified 1823 DEGs and 148 differentially expressed proteins (DEPs) from the decidual tissues of RM patients through transcriptomics and proteomics detection, respectively. Subsequently, five protein-encoding genes—*DCN, DPT, ISG15, LUM*, and *MFAP4*—were identified as RM-related hub genes through systematic bioinformatics analysis. The significantly increased decidual expression of these five genes was detected in RM patients, while an increased uterine expression of *Isg15* and decreased uterine expressions of *Dcn, Dpt, Lum*, and *Mfap4* were observed in LPS-induced EPL mice. Additionally, decreased *ISG15* expression was found to accompany the *in vitro* decidualization of hESCs.

Given that the decidua plays a critical role in embryo implantation and fetal development, abnormalities in decidualization and/or decidual functions could lead to RM. Thus, the same three pairs of decidual tissues collected from RM patients and normal early pregnant (NP) women were used to identify DEGs through RNA-seq analysis and DEPs through iTRAQ analysis. Although many DEGs and DEPs might not be the cause of RM, as the decidual tissues were not collected before the occurrence of miscarriage, the results of bioinformatics analysis could provide valuable insights into the potential molecular mechanisms underlying RM. Notably, these DEGs and DEPs were mainly associated with immunity, inflammation, and metabolic pathways. Specifically, GSEA analysis revealed that the top five upregulated pathways associated with these DEGs were similar to those associated with these DEPs, including allograft rejection, graft-versus-host disease, and rheumatoid arthritis, consistent with the concept that the embryo/fetus is a semi-allograft to the maternal organism.

Subsequently, five upregulated DEGs/DEPs—DCN, DPT, ISG15, LUM, and MFAP4—were selected as hub genes related to RM, based on their interactions with numerous other genes or regulatory elements in molecular networks, which are considered key factors in coordinating and balancing biological processes [25, 26]. The significantly increased decidual expression levels of these five genes in RM patients compared to those in normal early pregnant (NP) women were validated not only through qRT-PCR analysis but also by bioinformatics on a single-cell sequencing dataset GSE214607 from the GEO database. The subsequent analysis revealed that all five hub genes were highly expressed in DSCs and abnormally increased in the RM group. DCN, LUM, MFAP4, and DPT are all ECM components [27, 28]. It has been well demonstrated that ECM components are themselves signaling molecules that regulate cytokine activity and participate in driving various biological functions [29], and imbalances in the crosstalk between the ECM and the immune system could lead to various diseases [29]. As ISG15 is a key factor in the immune system [30], we speculated that ECM components (DCN, DPT, LUM, MFAP4) and ISG15 might play interdependent roles in the pathogenesis of RM.

Decorin (DCN), a type of ECM small leucine-rich proteoglycan (SLRP), was found to be expressed in chorionic villus mesenchymal cells and decidual cells during human pregnancy. Decidua-derived DCN inhibited the proliferation, invasion, and endovascular differentiation of trophoblasts [31]. It was reported that decidual DCN expression was increased in preeclampsia (PE) patients, and peripheral levels of both DCN and TNF-α were elevated in PE patients before the appearance of clinical signs, serving as predictive biomarkers for PE [31, 32]. Furthermore, DCN could promote the production of pro-inflammatory factors TNF-α and IL-12, and induce the M1 polarization of decidual macrophages (Mφs), ultimately contributing to RM occurrence [33, 34]. Consistent with these reported data, DCN was identified here as an RM-related hub gene, with increased decidual expression in RM patients.

Lumican (LUM), another SLRP, participates in regulating cell proliferation, differentiation, and inflammation [35]. During the peri-implantation period in mice, uterine expressions of DCN and LUM were detected in the stroma before decidualization; after the onset of decidualization, LUM was expressed in decidualized regions of the endometrial tissues, while DCN was absent. Embryo implantation failure was associated with decreased LUM expression in mice [36]. Additionally, it was reported that LUM promoted trophoblast proliferation, and decreased placental LUM expression might lead to PE [37]. These data suggest that increased LUM expression in ESCs and trophoblasts should be beneficial for pregnancy. We therefore speculated that the increased decidual LUM expression in RM patients observed here might result from its elevated expression in DICs.

Dermatopontin (DPT) is involved in the formation of extracellular architecture and adhesion and was detected in the bovine placenta during pregnancy and parturition. Its expression increased as pregnancy progressed, potentially regulating cell adhesion during attachment and detachment of the placenta in bovines [38]. In mice, uterine DPT expression increased dramatically during the pre-implantation period, reaching its highest level at implantation; decidualization did not affect uterine DPT expression, and DPT expression gradually decreased in the luminal epithelial cells, while it increased in ESCs as pregnancy advanced [39]. It was reported that in human adenocarcinoma cells, DPT expression was promoted by pro-inflammatory cytokines (LPS, TNFα, and TGFβ) but inhibited by the anti-inflammatory cytokine IL-4 [40].Thus, the increased decidual DPT expression in RM patients detected here might be due to enhanced inflammation at the maternal-fetal interface.

Microfibrillar-associated protein 4 (MFAP4) is a fibrinogen-related protein and plays an important role in ECM transformation during fibrogenesis [41]. In the absence of MFAP4, downregulation of pro-inflammatory cytokines (IL-1β and TNF-α) and concurrent inhibition of NF-κB signaling pathway activation can mitigate the inflammatory response induced by unilateral ureteral obstruction [42]. Recently, MFAP4 was identified as both an endometriosis-related [43] and an RM-related hub gene in immune cells [44], further supporting the conclusions of this study. However, the roles of MFAP4 in pregnancy remain unclear. As MFAP4 is also recognized as an integrin ligand with potential effects on pulmonary and vascular tissue homeostasis [45], we hypothesized that increased decidual MFAP4 expression might contribute to pregnancy loss by disrupting micro-homeostasis at the maternal–fetal interface.

Unexpectedly, only the uterine ISG15 expression was significantly increased, while uterine expression levels of DCN, DPT, LUM, and MFAP4 were significantly decreased in LPS-induced EPL mice. Furthermore, our lab [46] and another lab [47] previously reported that decidual ISG15 expression in RM patients was significantly elevated compared to NP women. Therefore, we focused mainly on ISG15, and the increased decidual ISG15 protein production in RM patients was further validated by Western blot analysis in this study.

Interferon-stimulated gene 15 (*ISG15*, also known as ubiquitin cross-reactive protein, UCRP), a 15-kDa ubiquitin-like protein, plays an important role in regulating various cellular functions through the modification of its target proteins via ISGylation [48, 49]. Here, we observed a significantly decreased *ISG15* expression after *in vitro* decidualization of T-HESCs, suggesting that increased *ISG15* expression may contribute to RM by affecting decidualization. However, it was reported that, as a component of the innate immune system, endometrial ISG15 expression was induced by pregnancy in mice and humans, and increased embryo mortality and smaller litter sizes were observed in ISG15-deficient (*Isg15*−/−) pregnant mice on day 12.5 postcoitum [50]. Additionally, increased *ISG15* expression within stromal cells undergoing decidualization was detected in human decidual tissues during the first trimester [51]. Given that RM is associated with elevated levels of pro-inflammatory cytokines IL-1β and TNFα, as well as M1 polarization of decidual macrophages [19], and ISG15 can induce IL-1β and TNFα expression and promote M1 macrophage polarization through ISGylation of STAT1 [52], increased decidual ISG15 expression may contribute to RM by promoting M1 macrophage polarization through dysregulated ISGylation. Consistently, ISG15 was reported to have inhibitory effects on female fertility through ISGylation [53].

Additionally, miR-27a-3p, miR-16-5p, miR-21-3p, and miR-941 were identified as potential upstream regulators of these five hub genes, and significantly decreased decidual expression levels of these four miRNAs were observed in RM patients as expected. It was reported that miR-27a-3p expression was detected in human decidual tissues during early pregnancy [54], and upregulation of miR-27a-3p could inhibit the expression of IL-1β, TNFα, and IL-6 through the TLR4/NF-κB pathway [55, 56]. Abnormally downregulated miR-16-5p expression was found to be associated with maternal pathophysiology [57], and the deficiency of miR-16-5p in vascular cells led to increased expressions of IL-1β and TNFα [58]. Notably, decreased expressions of miR-21-3p and miR-941 were associated with preeclampsia [59, 60]. Thus, the reduced expression of miR-27a-3p, miR-16-5p, miR-21-3p, and miR-941 might be involved in RM pathogenesis by leading to the increased expression of *DCN, DPT, ISG15, LUM*, and *MFAP4* at the maternal–fetal interface.

Finally, based on the DSigDB database, we preliminarily explored potential drugs for treating RM that target these five identified hub genes. The results indicate that different drugs exhibit varying specificity toward these hub genes, with retinoic acid (RA) targeting all five. RA and its derivatives, such as isotretinoin and acitretin, have demonstrated efficacy and safety across a wide range of clinical applications [61]. Another study suggested a ROS-mediated mechanism through which RA regulates localized VEGF secretion in the human endometrium, potentially essential for the successful establishment of pregnancy [62]. Based on these findings, RA appears to be a promising therapeutic intervention for future research in reproductive health.

It should be noted that this study has limitations. We focused only on genes (proteins) that exhibited similar trends in both omics datasets, but genes and proteins with opposing expression trends between the two datasets may be involved in more complex post-transcriptional regulation mechanisms. Moreover, it is noteworthy that all the identified hub genes showed increased decidual expression levels in RM patients. However, based on our pathway enrichment analysis, downregulated genes potentially play important roles in glucose and lipid metabolism. Furthermore, the small sample size of human decidual tissues might have led to the discovery of relatively few differential genes/proteins. Therefore, the data presented here should be considered preliminary results, as in our previous study [63]. Additionally, as decidual tissues were collected from RM patients and NC women after pregnancy termination, the differential expression of identified hub genes in RM patients might be an effect of the miscarriage rather than its cause. In fact, most RM-related omics studies face similar limitations due to the difficulty of collecting decidual or villus tissues from RM patients and normal early pregnant women [63–65]. Moreover, the decidual samples used in this study were collected from only one clinic site, limiting the generalizability of the results given the differences in genetic characteristics, lifestyles, and climatic conditions across regions. Although numerous studies have identified DEGs in decidual and villus tissues of RM patients, few genes have been consistently identified across studies. However, we believe that each individual omics study can contribute to a more comprehensive understanding of RM-related DEGs, and bioinformatics and big data tools will expedite this process.

## Conclusion

*DCN, DPT, ISG15, LUM,* and *MFAP4* were identified as RM-associated hub genes, while hsa-miR-16-5p, miR-21-3p, miR-27a-3p, and miR-941 were identified as upstream regulators of these hub genes. The decidual expression levels of these five hub genes were increased, whereas those of these four miRNAs were decreased in RM patients. Uterine *Isg15* expression was also elevated in LPS-induced EPL mice, and the *in vitro* decidualization of human ESCs was accompanied by reduced *ISG15* expression. Retinoic acid (RA) was found to target all five hub genes. These findings present potential biomarkers, targets, and drug candidates for RM treatment.

## Supplemental data

**Table S1:** Sequences of primers for qPCR analysis

**Primer name**	**Sequence (5′ to 3′)**
Homo ISG15 Forward	TGGACAAATGCGACGAACCTC
Homo ISG15 Reverse	TCAGCCGTACCTCGTAGGTG
Homo LUM Forward	CTGCGTTTATCTCACAACGAACT
Homo LUM Reverse	CAGATCCAGCTCAACCAGGG
Homo DPT Forward	TGGGTGAATTTGAACCGGCAA
Homo DPT Reverse	CGTAGTTCCATTGTCTGTCAGAA
Homo DCN Forward	GACAACAACAAGCTTACCAGAGTA
Homo DCN Reverse	TGAAAAGACTCACACCCGAATAAG
Homo MFAP4 Forward	CGGCTGGAATGACTACAAG
Homo MFAP4 Reverse	CAGTGTCAGGAGGTGCATG
Homo actin Forward	CATGTACGTTGCTATCCAGGC
Homo actin Reverse	CTCCTTAATGTCACGCACGAT
Homo RPL7 Forward	CAAGGCTTCGATTAACATGCTGA
Homo RPL7 Reverse	GCCATAACCACGCTTGTAGATT
Homo PRL Forward	CTACATCCATAACCTCTCCTCAG
Homo PRL Forward	GGGCTTGCTCCTTGTCTTC
Homo IGFBP1 Forward	AGAGTCGTAGAGAGTTTAGC
Homo IGFBP1 Reverse	ACACTGTCTGCTGTGATAA
Mouse ISG15 Forward	TGGTACAGAACTGCAGCGAG
Mouse ISG15 Reverse	AGCCAGAACTGGTCTTCGTG
Mouse LUM Forward	ATAGTGGGGTACCTGGAAACTCGT
Mouse LUM Reverse	CCAGGATCTTACAGAAGCTCTTCA
Mouse DPT Forward	GGATCGTGAGTGGCAATTTT
Mouse DPT Reverse	CGAATTCGCAGTCGTAGTCA
Mouse DCN Forward	GAGGGAACTCCACTTGGACA
Mouse DCN Reverse	TTGTTGTTGTGAAGGTAGACGAC
Mouse MFAP4 Forward	GCAACCCCTGGACTGTGATG
Mouse MFAP4 Reverse	TTGTCATGTCGCAGAAGACGG
Mouse GAPDH Forward	ACCCAGAAGACTGTGGATGG
Mouse GAPDH Reverse	TTCAGCTCAGGGATGACCTT

qPCR: Quantitative PCR; LUM: Lumican; DPT: Dermatopontin; DCN: Decorin; MFAP4: Microfibrillar-associated protein 4.

**Table S2:** Characteristics of recruited RM patients and NC women at early pregnancy for RNA-seq and iTRAQ analyses

	**RM patients**	**NC women**	***P* value**
*n*	3	3	N/A
Age (years)	31.00 ± 2.76	30.33 ± 4.84	>0.05
Gestational weeks	8.67 ± 0.52	8.00 ± 0.89	>0.05
Gravidity	2.33 ± 0.52	2.2 ± 1.2	>0.05
Parity	0 [0, 0]	0 [0, 0]	>0.05
Miscarriage history	2 [2, 3]	0 [0, 0]	<0.05

RM: Recurrent miscarriage; NC: Normal control; RNA-seq: RNA sequencing.

**Table S3:** Characteristics of recruited RM patients and NC women at early pregnancy for RT-qPCR analysis

	**RM patients**	**NC women**	***P* value**
*n*	9	9	N/A
Age (years)	29.33 ± 3.67	31.75 ± 5.87	>0.05
Gestational weeks	9.44 ± 1.33	8.38 ± 1.92	>0.05
Gravidity	2.78 ± 0.67	2.50 ± 1.07	>0.05
Parity	0 [0, 0.5]	1 [0, 1.8]	>0.05

RM: Recurrent miscarriage; NC: Normal control; RT-qPCR: Real-time quantitative PCR.

**Table S4:** Predicted top ten drugs targeting the RM-related hub genes

**Drug name**	***P* value**	**Combined score**	**Related genes**
Testosterone enanthate	1.81E–04	474.7605252	*LUM; DPT; ISG15*
Cytarabine	2.49E–04	408.6156267	*MFAP4; LUM; DCN*
Retinoic acid	4.37E–04	608943.1725	*MFAP4; LUM; DPT; DCN; ISG15*
L-leucine	8.09E–04	522.5218396	*LUM; DCN*
Mifepristone	0.002689455	233.682378	*LUM; DCN*
Tetradioxin	0.005343715	90.24218974	*MFAP4; LUM; ISG15; DCN*
Dasatinib	0.005979206	133.0720253	*MFAP4; LUM*
Mefloquine	0.007229642	878.8271482	*ISG15*
Chlorophyllin	0.007478193	842.6622961	*ISG15*
Prenylamine	0.009464812	628.4308111	*ISG15*

RM: Recurrent miscarriage.

## Data Availability

The data that support the findings of this study are available from the corresponding author upon reasonable request.
